# Clinical Prognostic Factors in Patients With Metastatic Adrenocortical Carcinoma Treated With Second Line Gemcitabine Plus Capecitabine Chemotherapy

**DOI:** 10.3389/fendo.2021.624102

**Published:** 2021-02-24

**Authors:** Salvatore Grisanti, Deborah Cosentini, Marta Laganà, Alessandra Morandi, Barbara Lazzari, Laura Ferrari, Alberto Dalla Volta, Roberta Ambrosini, Vittorio Domenico Ferrari, Sandra Sigala, Alfredo Berruti

**Affiliations:** ^1^ Medical Oncology Unit, Department of Medical and Surgical Specialties, Radiological Sciences and Public Health, University of Brescia at ASST Spedali Civili, Brescia, Italy; ^2^ Radiology Unit, Azienda Socio Sanitaria Territoriale (ASST) Spedali Civili, Brescia, Italy; ^3^ Section of Pharmacology, Department of Molecular and Translational Medicine, University of Brescia, Brescia, Italy

**Keywords:** adrenocortical carcinoma, gemcitabine, capecitabine, prognostic factor, chemotherapy

## Abstract

Gemcitabine plus Capecitabine (Gem/Cape) is a frequently adopted second line chemotherapy for metastatic adrenocortical carcinoma (ACC), but only a minority of patients is destined to obtain a clinical benefit. The identification of baseline predictive factors of efficacy is relevant. We retrospectively analyzed clinical data from 50 consecutive patients with metastatic progressing ACC treated between 2011 and 2019. Patients received intravenous Gemcitabine and oral Capecitabine on a metronomic schedule. Previous mitotane therapy was maintained. Clinical benefit (partial response + stable disease) at 4 months was 30%, median progression-free survival (PFS) and disease-specific survival (DSS) from Gem/Cape start were 3 and 8 months, respectively. Among clinical variables evaluated before the start of Gem/Cape, presence of ECOG performance status ≥1 [HR 6.93 95% confidence interval (CI) 0.03–0.54, p.004] and neutrophil-to-lymphocyte ratio (NLR) ≥5 [HR 3.88, 95% (CI) 0.81–0.90, p.003] were independent indicators of poor PFS at multivariate analysis. Conversely, surgery of primary tumor, the presence of lung or lymph-node metastases, blood mitotane level, anemia, and the Advanced Lung cancer Inflammation index (ALI) failed to be independently associated. This study confirms that the Gem/Cape schedule is modestly active in heavily pretreated ACC patients (28% received at least two previous chemotherapy lines). NLR and performance status (PS) are easily available clinical parameters that are helpful to identify patients not likely to derive significant advantage from Gem/Cape chemotherapy.

## Introduction

The clinical management of patients with metastatic adrenocortical carcinoma (ACC) remains a challenging issue for a number of reasons. First, while there are patients with a relatively indolent disease that can be controlled by either mitotane monotherapy or locoregional treatments, the majority of cases displays more aggressive and highly proliferating disease that requires the adoption of systemic chemotherapy (1, 2). Second, according to the results of the FIRM-ACT trial the current first line chemotherapy for locally advanced or metastatic ACC, namely the EDP-M schedule, offers a median progression-free survival (PFS) of 5.1 months (3). Although there is a small proportion of long lasting responder patients, this median PFS indicates that approximately 50% of patients will require additional treatments in the 6 months following EDP failure (4). Third, the EDP regimen administration is associated to manageable but consistent toxicities which causes not all patients can be treated with full dose intensity because of age, performance status (PS), or comorbidity limitations. These patients will require less intensive, alternative schedules following EDP. Fourth, because of its rarity, ACC is an orphan cancer in terms of new drugs selection and thus, very few options exist after failure of standard first-line chemotherapy and the reported overall response rate and duration of response are exceedingly dismal (5–8).

In 2005, based on evidence derived from single case reports, an international consensus conference suggested to incorporate gemcitabine [20,20-difluorodeoxycytidine (Gem)] as a promising agent in the treatment of ACC (9). In a phase 2 clinical trial, 28 patients with advanced ACC were treated with Gem combined with fluoropyrimidines [capecitabine (Cape) or 5-fluorouracil (5FU)] (10). In this pilot study, the overall response rate (CR+PR) was 7%, the disease control rate (CR+PR+SD) was 46.3% and the median progression-free survival was 5.3 months. In a retrospective analysis of a German and Italian series of 145 advanced ACC patients treated outside a clinical trial, Gem/Cape chemotherapy was associated with a disease control rate in approximately 30% of patients and a clinical benefit (disease stabilization or response to therapy for ≥4 months) in approximately 20% of patients (11). These results confirmed that this regimen was moderately active and well tolerated in the real-world practice. To date there is a general consensus in using Gem-Cape chemotherapy as a second line approach in ACC patients after failure of EDP (1).

A central issue in the management of patients with advanced cancers is the preservation of quality of life (QOL) (12). Thus, the prescription of a palliative treatment should be guided by factors enabling the clinician to pre-select the potential responder patient avoiding unnecessary toxicity to the others (13, 14). However, while several studies focused on identification of clinical and pathological indicators (such as the GRAS parameters in the modified ENSAT classification) as adjunct prognostic factors in the setting of advanced ACC (15), less attention has been paid to select patients that have a realistic chance of obtaining benefit from chemotherapy. To address this issue, we analyzed a series of patients with advanced ACC treated with Gem and Cape with the aim of identifying clinical characteristics and laboratory parameters that can easily be found in the daily clinical practice, to be used as predictive factors of efficacy.

## Materials and Methods

### Study Design, Patients Selection, and Treatment

This is a retrospective analysis of consecutive patients treated outside a clinical trial at the Medical Oncology Department of the Spedali Civili of Brescia. The study was approved by the Institutional Ethical Review Board (ID: NP 3776/2019) and conducted in accordance with the principles of the Declaration of Helsinki. The decision of starting Gem/Cape chemotherapy was taken within the ACC tumor board discussion for each single case. Patients were included in this retrospective analysis with the following main inclusion criteria: histologically confirmed diagnosis of ACC; clinical, biochemical, and radiological evidence of disease progressing after first–line treatment and not eligible of loco-regional therapies; measurable disease; life expectancy of at least 3 months, age ≥18 years; ECOG performance status (ECOG PS) 0-2; adequate organ function; ability to sign an informed consent.

Main exclusion criteria were: previous treatment with gemcitabine and/or fluoropyrimidines; known hypersensitivity to gemcitabine and/or fluoropyrimidines; history of previous neoplasm within 5 years. The primary objective was to identify clinical and biochemical indicators predictive of response to therapy in terms of progression-free survival (PFS). Secondary endpoints were best objective response, toxicity and disease-specific survival (DSS) analysis from Gem/Cape chemotherapy.

Clinical and biochemical parameters were calculated both at initial diagnosis of ACC and before initiation of Gem/Cape chemotherapy. They included: sex, age, medical history pre- and post-Gemcitabine treatment, ECOG performance status (PS), and the Charlson’s Comorbidity Index (CCI) score (16), routine laboratory tests measured at baseline including the mitotane plasma levels, absolute count of white blood cells (WBC), relative counts of neutrophils and lymphocytes, hemoglobin value, platelets absolute count, and the neutrophils-to-lymphocytes ratio (NLR) categorized as <5 or ≥5, derived NLR {dNLR: calculated as [neutrophil count/(leucocyte-neutrophil count)]} (17).

In addition, we assessed the Lung Immune Prognostic Index (LIPI index) which combined the dNLR and LDH levels and categorized patients into three prognostic subsets based on the following cutoffs: dNLR ≤ 3 and LDH ≤UNL; dNLR ≥3 or LDH≥ULN; dNLR≥3 and LDH≥ULN which defined good, intermediate, poor prognostic groups, respectively (17).

The Advanced Lung cancer Inflammation index (ALI index) was calculated as BMI × ALB/NLR. Patients were divided into the low-ALI and high-ALI groups according to the median value (18).

The treatment schedule was based on the regimen previously published by Sperone et al. (10). Briefly, Gem was administered at 800 mg/sqm over 30 min. intravenous infusion on days 1 and 8 every 21 days, and oral Cape was given at 1500 mg/daily continuously. All patients received concomitant mitotane with the goal of maintaining the 14–20 mg/L target plasma concentration. Chemotherapy was continued until disease progression or unacceptable toxicity. Dose reductions or withdrawal of one drug was permitted according to the clinician’s decision.

### Evaluation of Response and Toxicity

Minimum work-up at baseline included physical examination, biochemical and hormonal assessment, and tumor-parameters assessed by imaging techniques not older than 6 weeks. Imaging included total body computed tomography (CT) or FdG-positron emission tomography (CT/PET) or regional nuclear magnetic resonance (MNR). Biochemical and hormonal profiles were assessed at the beginning of each cycle. Radiological assessment was repeated every 12 weeks with the same imaging parameter technique and evaluation of response was classified according to RECISTv1.1 criteria as complete response (CR), partial response (PR), stable disease (SD), or progressive disease (PD). Clinical benefit was defined as stable disease (or better response) lasting at least 4 months. Toxicities were registered during treatment and follow-up and were graded using the Common Terminology Criteria for Adverse Events (CTCAE), version 4.03.

### Statistical Analysis

Descriptive statistics were used to analyze clinical indicators. Continuous variables were categorized by identification of optimal cut-off values. Associations between categorical variables were assessed by two-sided chi-square or Fisher tests as appropriate. PFS and DSS were calculated as the time elapsed from start of Gem/Cape to the first radiological evidence of PD or to the date of death related to ACC, respectively. Survival curves were generated with the Kaplan–Meier method and compared with the log-rank test (Mantel-Cox). Clinical variables with a potential prognostic value at univariate Cox regression (enter level p ≤.05) were included in the multivariate Cox model. Results are given as hazard ratio (HR) with 95% confidence intervals (95% CIs). A Bonferroni correction was applied in the multivariate Cox model to correct for multiple comparisons with a small sample size and the new significance level was set at p.006. For all other tests the statistical significance was conventionally set at p <.05. All analyses were performed with Statistical Package for Social Science (SPSS Software, Version 23.0, IBM SPSS Statistics, Chicago, IL, USA).

## Results

### Patients Clinicopathological Characteristics, Treatment, and Toxicity

From January 2011 to December 2019, 50 patients with advanced ACC were sequentially treated with Gem/Cape chemotherapy. Patients characteristics are summarized in **Table 1**. At initial diagnosis 50% of the patients had an ENSAT stage IV tumor and 52% had hormonal hypersecretion, respectively. The majority (84%) of patients underwent surgery that was radical (R0) in less than 30% of them. Median Ki67 proliferation index was 25%. All patients received postoperative mitotane therapy. Disease progression/relapse occurred in both visceral and non-visceral anatomical sites in 64% of patients and the most frequent anatomic sites were the lungs, liver, extra-liver abdominal sites and lymphnodes. The most frequent metastatic pattern was represented by lung, liver and abdominal lesions in 26% of patients. The vast majority of patients (70%) had received at least one previous line of chemotherapy for advanced disease and the median time from diagnosis to Gem/Cape chemotherapy was 19 months. Before starting Gem/Cape, more than 60% of the patients had disease-related symptoms (ECOG PS ≥1) and displayed a Charlson’s Comorbidity Index score≥5 indicating the presence of at least 3 significant comorbidities. Ten (20%) patients displayed clinical signs of steroid excess. Mitotane levels were available for 45 (90%) patients and 64% of them had a mitotane concentration below the therapeutic range. Laboratory abnormalities showed anemia (Hb <12 g/dl) in 61% of patients while total leukocytes and platelets were within normal range in 27 (66%) and 23 (64%) of cases, respectively. NLR above 5 was observed in 21 (42%) cases. Eight patients (16%) without baseline LDH or dNLR were excluded from the LIPI index calculation. Among the 42 (84%) evaluable patients, 16 (38%) had good LIPI, and 25 (59%) had intermediate-poor LIPI. We additionally calculated the ALI index in the 50 patients in which NLR, BMI, and ALB were available: 25 patients (50%) had an ALI index below-equal the median value (40), the remaining 25 had greater values. No other biochemical parameters are reported because of missing values in >50% of cases.

**Table 1:** Patients clinical and pathological characteristics at initial diagnosis and at start of Gem/Cape chemotherapy.

Characteristic	N. ACC patients (%)
Median age (range), years	49 (16–68)
<50	26/50 (52)
≥50	24/50 (48)
Sex	
Male	22/50 (44)
Female	28/50 (56)
Initial ENSAT tumor stage:	
I-II III	16/50 (32) 9/50 (18)
	
IV	25/50 (50)
Hormone secretion:	
No secretion	24/50 (48)
Hormone hypersecretion	26/50 (52)
Glucocorticoids	22/26 (85)
Androgens	3/26 (11)
Aldosterone	1/26 (4)
Pathology	
Median Ki67 (range), %	25 (5–85)
Median Weiss score (range)	6 (3–9)
Surgery and resection status:	
No surgery	8/50 (16)
R0	12/42 (28)
RX	4/42 (10)
R1/R2	26/42 (62)
Number of previous chemotherapy lines 1	36/50 (72)
	
≥2	14/50 (28)
Time from ACC diagnosis to Gem/Cape start	
<19	24/47 (51)
≥19	23/47 (49)
Types of previous chemotherapy lines	
Cisplatin-based	31/50 (62)
Anthracyclines-based	30/50 (60)
Streptozotocin/Temozolomide	4/50 (8)
Mitotane concentration during Gem/Cape chemotherapy	
<14 mg/L	29/45 (64)
14–20 mg/L	16/45 (36)
Unknown	5/50 (18)
ECOG performance status at Gem/Cape start	
0	19/50 (38)
≥1	31/50 (62)
Charlson’s Comorbidity Index (CCI) at Gem/Cape start	
Score 0–2	14/45 (31)
Score 3–5	6/45 (13)
Score ≥ 6	25/45 (56)
Unknown	5/50 (10)
Metastatic sites at Gem/Cape start	
Lung	38/50 (76)
Abdomen/peritoneum	35/50 (70)
Liver	30/50 (60)
Lymphnodes	17/50 (34)
Bone	2/50 (4)
White blood cells (WBC) at Gem/Cape start	
<4 × 10^3^/μl	9/41 (22)
4–10.8 × 10^3^/μl	27/41 (66)
>10.8 × 10^3^/μl	5/41 (12)
Unknown	9/50 (18)
Lactate dehydrogenase (LDH) at Gem/Cape start	
Median LDH (range)	220 (123-1867)
≤225 U/L	21/42 (50)
>225 U/L	21/42 (50)
Unknown	8/50 (16)
Albumin (ALB) at Gem/Cape start	
Median ALB (range) ≤3.5 g/dl	3.5 (2–4.6) 28/50 (56)
>3.5 g/dl	22/50 (44)
Body mass index (BMI)	
Median ALB (range)	23.5 (17.8–34.4)
≤23.5	26/50 (52)
>23.5	24/50 (48)
Hemoglobin (Hb) at Gem/Cape start	
≤12 g/dl	25/41 (61)
>12 g/dl	16/41 (39)
Unknown	9/50 (18)
Platelet (PLT) at Gem/Cape start	
<130 × 10^3^/μl	1/36 (3)
130 – 400 × 10^3^/μl	23/36 (64)
>400 × 10^3^/μl	12/36 (33)
Unknown	14/50 (28)

All patients received combination chemotherapy with both Gem and Cape. A median number of 3 cycles (range 1–17) was administered and the median dose intensity (median mg/sqm administered/mg/sqm calculated per cycle) of Gem and Cape was 93 and 100%, respectively. Treatment was overall well tolerated with all grades asthenia in 33% of patients and grade 3–4 adverse events being reported in less than 10% of patients. In this subgroup of patients, CTCAE grade 3–4 anemia and neutropenia were the most frequent adverse events.

### Response to Therapy and Survival Analysis

**Figure 1:**
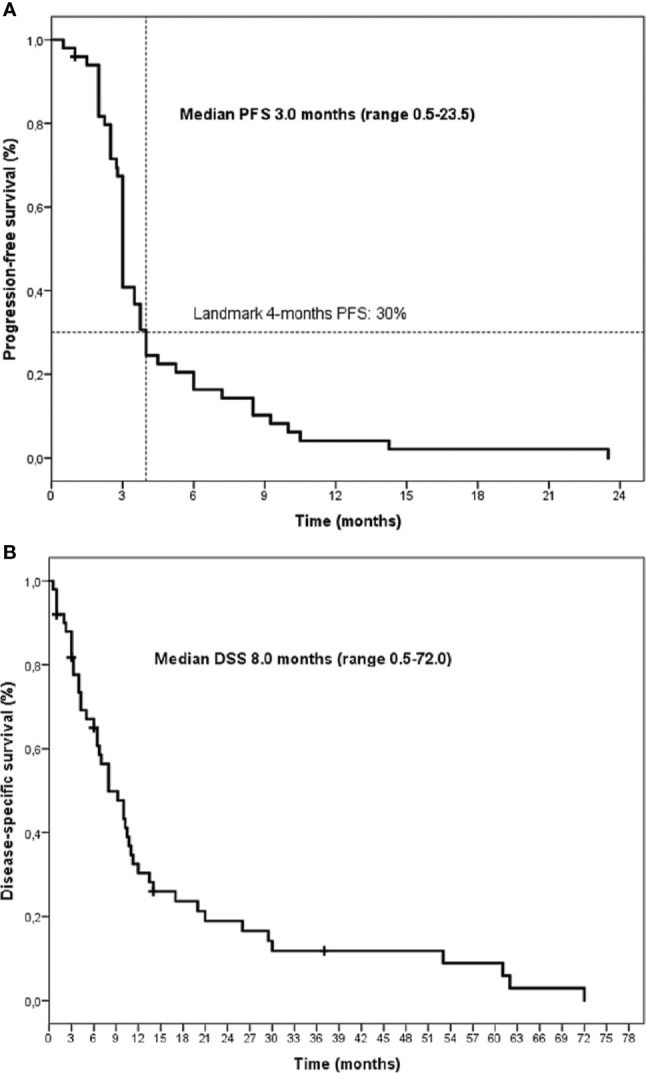
Kaplan-Meier curves of progression-free survival (PFS) **(A)** and disease-specific survival (DSS) **(B)** for the whole series.

All patients were eligible for response and survival. After a median follow-up of 8 months (last update December 2019), all patients but one had progressed with a median PFS of 3 months (range 0.5–23.5) and 45/50 (90%) were dead with a median DSS of 8 months (range 0.5–72) (**Figure 1**). No complete responses were observed. Analysis of best response included 7 (14%) partial responses and 12 (24%) stable diseases. In a landmark analysis at 4 months from Gem/Cape start, 30% of patients obtained a clinical benefit (PR+SD) (**Table 2**). Clinical benefit was however transient and 18% and 4% of patients were progression-free at 6 and 12 months, respectively. After progression, 17 (34%) and 7 (14%) patients further received 1 and ≥2 lines of chemotherapy, respectively.

**Table 2:** Analysis of activity of Gem/Cape chemotherapy.

Endpoints of survival and response to therapy	Outcome
Median PFS (range), months	3.0 (0.5–23.5)
Median DSS (range), months	8 (0.5–72)
Best objective response to therapy:	
Progressive disease (PD)—n (%)	31/50 (62)
Stable disease (SD)—n (%)	12/50 (24)
Partial response (PR)—n (%)	7/50 (14)
Clinical benefit at 4 months (PR+SD)—n (%)	15/50 (30)
Clinical benefit at 6 months (PR+SD)—n (%)	9/50 (18)
Clinical benefit at 12 months (PR+SD)—n (%)	2/50 (4)

### Analysis of Predictive and Prognostic Factors

Two sets of variables were evaluated: traditional prognostic clinico-pathological variables (including ENSAT stage, Ki67, resection status, hormonal hypersecretion) at initial diagnosis and patients clinical characteristics before the start of Gem/Cape chemotherapy (including ECOG PS, comorbidities, pattern of metastases distribution) and laboratory abnormalities. All sets of variables were tested for their predictive and prognostic value in terms of PFS and DSS. Included variables are summarized in **Tables 3** and **4**. Among the variables at initial diagnosis only no-frontline surgery of the primary tumor resulted predictive of shorter PFS during Gem/Cape therapy at univariate analysis (p.014). Clinical variables evaluated before the start of Gem/Cape associated with a higher risk of progression at univariate analysis included: ECOG PS ≥1 (p <.0005), lung metastases (p.040), lymph-node metastases (p.015), neutrophils **>**70% (p.015), lymphocytes **<**20% (p.009), neutrophils/lymphocytes ratio (NLR) ≥5 (p.015), Hb **<**12 g/dl (p.017), ALI index lower than 40 (median value) (p.038), and low mitotane concentrations during Gem/Cape chemotherapy (p.036). At multivariate analysis, no-frontline surgery (HR 2.67, 95% CI 1.00–7.10, p.049), presence of symptoms and pain (ECOG PS ≥1) (HR 6.93, 95% CI 1.86–25.79, p.004) and NLR **>**5 (HR 3.88, 95% CI 1.57–9.54, p.003) remained significant as independent predictors of poorer PFS. Finally, after Bonferroni correction only ECOG PS ≥1 and NLR **>**5 maintained a statistical significance (p <.006). Mitotane plasma concentrations and the metastatic sites pattern did not show a prognostic value either for PFS or for DSS. When looking at variables with potential impact on DSS, ENSAT stage IV at initial diagnosis (p.049), time-to Gem/Cape start **<**19 months (p.008), ECOG PS ≥1 (p.012), and low mitotane concentrations during Gem/Cape chemotherapy (p.037) were significant at univariate analysis. However, none of these covariates maintained a statistical significance at multivariate analysis.

**Table 3:** Uni- and multivariate analysis of clinico-pathological factors predictive of progression-free survival (PFS).

Variable		Univariate analysis				Multivariate analysis		
		Median PFS (mo)	HR	95% CIs	p	HR	95% CIs	p
Age at diagnosis	<50 ≥50	3.00 3.00	1.00 1.16	.65–2.07	.597	–	–	–
Sex	Female Male	3.00 3.00	1.00 1.02	.57–1.82	.942	–	–	–
ENSAT stage at diagnosis	I–III IV	3.00 3.00	1.00 1.48	.83–2.64	.177	–	–	–
Hormone hypersecretion	No Yes	3.00 3.00	1.00 1.01	.57–1.78	.971	–	–	–
Proliferation index (Ki67)	≤20% >20%	4.00 3.00	1.00 1.36	.72–2.54	.336	–	–	–
Surgery of primary tumor	No Yes	3.00 2.50	1.00 2.72	1.22–6.05	.014	2.67	1.00–7.09	.049
Previous lines of CT	1 ≥2	3.00 3.00	1.00 1.33	.74–2.37	.331	–	–	–
Time from ACC diagnosis to Gem/Cape start	<19 ≥19	3.00 3.00	.479	.257–0.89	.121	–	–	–
ECOG performance status at Gem/Cape start	0 ≥1	6.00 3.00	1.00 4.83	2.19–10.66	<.0005	.145	.03–0.54	.004
Charlson’s Comorbidity Index at Gem/Cape start	Score 0–5 Score ≥6	3.00 3.50	1.00 1.02	.56–1.88	.928	–	–	–
Metastases lung	No Yes	6.00 3.00	1.00 2.12	1.03–4.37	.040	.837	.24–2.82	.77
Metastases liver	No Yes	3.50 3.00	1.00 1.78	.96–3.32	.066	–	–	–
Metastases lymphnodes	No Yes	3.00 2.80	1.00 2.21	1.16–4.22	.015	.551	.23–1.3	.17
Metastases abdomen/peritoneum	No Yes	3.00 3.00	1.00 1.04	.55–1.94	.902	–	–	–
Mitotane concentration during Gem/Cape chemotherapy	14–20 mg/L <14 mg/L	3.50 3.00	1.00 1.99	1.04–3.81	.036	1.25	.48–3.28	.64
Leukocytes (WBC) absolute count at Gem/Cape start	<4 × 10^3^/μl 4–10 × 10^3^/μl >10 × 10^3^/μl	3.00 3.00 2.00	.795 1.00 1.25	.26–2.42 .30–1.47 .41–3.81	.686 .318 .686	–	–	–
Neutrophils relative count at Gem/Cape start	<40–70% >70%	3.00 2.50	1.00 2.65	1.21–5.81	.015	–	–	–
Lymphocyte relative count at Gem/Cape start	<20% 20–>45%	2.50 3.00	1.00 2.81	1.29–6.12	.009	–	–	–
Albumine (ALB) at Gem/Cape start	>3.5 g/dl ≤3.5 g/dl	3.00 3.00	1.00 1.71	.92–3.17	.089			
Lactate dehydrogenase (LDH) at Gem/Cape start	≤225 U/L >225 U/L	3.00 3.00	1.00 1.65	.88–3.11	.116			
Body Mass Index BMI	≤23.5 >23.5	3.00 3.00	1.00 1.23	.67–2.24	.492			
Neutrophil-to-Lymphocyte Ratio (NLR) at Gem/Cape start	<5 ≥5	3.00 2.50	1.00 2.65	1.21–5.81	.027	.270	0.81–0.90	.003
Hemoglobin (Hb) at Gem/Cape start	≤12 g/dl >12 g/dl	3.00 3.00	2.44 1.00	1.17–5.09	.017	.804	.31–2.03	.646
Platelet (PLT) absolute count at Gem/Cape start	<130×10^3^/μl 130–400×10^3^/μl >400×10^3^/μl	2.25 3.00 3.00	3.78 .95 .26	.44–31.87 .46–1.97 .03–2.22	.221 .903 .221	–	–	–
LIPI index	Good prognosis Intermediate-poor prognosis	3.50 3.00	1.00 1.31	.68–2.52	.413	–	–	–
ALI index	>40 ≤40	3.00 3.00	1.00 1.90	1.03–3.50	.038	.932	.29–2.98	.906

**Table 4:** Uni- and multivariate analysis of clinico-pathological factors prognostic of disease-specific survival (DSS).

Variable		Univariate analysis				Multivariate analysis		
		Median DSS (mo)	HR	95% CIs	p	HR	95% CIs	p
Age at diagnosis	<50 ≥50	10.00 8.00	1.00 1.28	.70–2.25	.413	–	–	–
Sex	Female Male	8.00 10.25	1.00 1.38	.75–2.54	.292	–	–	–
ENSAT stage at diagnosis	I–III IV	10.25 6.50	1.00 1.86	1.00–3.47	.049	1.19	.56–2.84	.680
Hormone hypersecretion	No Yes	10.25 8.00	1.00 1.26	.69–2.30	.434	–	–	–
Proliferation index (Ki67)	≤20% >20%	10.75 8.00	1.00 1.32	.68–2.53	.402	–	–	–
Surgery of primary tumor	No Yes	10.00 4.25	1.00 1.81	.79–4.14	.158	–	–	–
Previous lines of CT	1 ≥2	9.25 8.00	1.00 1.02	.55–1.88	.942	–	–	–
Time from ACC diagnosis to Gem/Cape start	≥19 <19	13.5 6.5	1 0.40	.20–0.78	.008	.55	.22–1.40	.214
ECOG Performance Status at Gem/Cape start	0 ≥1	13.50 6.00	1.00 2.40	1.20–4.77	.012	1.93	.75–4.95	.167
Charlson’s Comorbidity Index at Gem/Cape start	Score 0–5 Score ≥6	9.25 8.00	1.00 1.82	.91–3.61	.086	–	–	–
Metastases lung	No Yes	13.50 7.00	1.00 1.99	.96–4.12	.064	–	–	–
Metastases liver	No Yes	11.00 7.00	1.00 1.73	.89–3.34	.103	–	–	–
Metastases lymphnodes	No Yes	10.25 5.00	1.00 1.60	.85–3.03	.143	–	–	–
Metastases abdomen/peritoneum	No Yes	8.00 9.25	1.00 .89	.46–1.71	.733	–	–	–
Mitotane concentration during Gem/Cape chemotherapy	14–20 mg/L <14 mg/L	12.00 7.00	1.00 2.12	1.04–4.29	.037	1.10	.44–2.74	.837
Leukocytes (WBC) absolute count at Gem/Cape start	<4 × 10^3^/μl 4–10 × 10^3^/μl >10 × 10^3^/μl	4.25 8.00 8.00	2.45 1.20 .40	.66–9.13 .35–4.08 .11–1.51	.180 .761 .180	–	–	–
Neutrophils relative count at Gem/Cape start	<40–70% >70%	8.00 6.50	1.00 1.44	.63–3.27	.377	–	–	–
Lymphocyte relative count at Gem/Cape start	<20% 20–>45%	6.50 9.25	1.00 1.52	.68–3.36	.301	–	–	–
Albumine (ALB) at Gem/Cape start	>3.5 g/dl ≤3.5 g/dl	10.25 8.00	1.00 1.44	.78–2.68	.242			
Lactate dehydrogenase (LDH) at Gem/Cape start	≤225 U/L >225 U/L	10.00 6.50	1.00 1.40	.71–2.73	.324			
Body mass index BMI	>23.5 ≤23.5	9.20 6.70	1.00 1.15	.61–2.18	.651			
Neutrophil/lymphocyte ratio (NLR) at Gem/Cape start	<5 ≥5	8.00 6.50	1.00 1.44	.63–3.27	.377	–	–	–
Hemoglobin (Hb) at Gem/Cape start	≤12 g/dl >12 g/dl	6.75 10.75	1.92 1.00	.92–4.00	.078	–	–	–
Platelet (PLT) absolute count at Gem/Cape start	<130×10^3^/μl 130–400×10^3^/μl >400×10^3^/μl	7.00 10.00 6.75	1.07 .49 .92	.13–8.57 .21–1.12 .11–7.40	.945 .093 .945	–	–	–
LIPI index	Good prognosis Intermadiate-poor prognosis	9.25 6.75	1.00 1.02	.52–2.00	.943	–	–	–
ALI index	>40 ≤40	10.00 6.50	1.00 1.41	.78–2.57	.252	–	–	–

## Discussion

In this paper we performed a retrospective analysis of ACC patients treated with Gem/Cape chemotherapy outside a clinical trial. This series is representative of patients treated with second-line chemotherapy for metastatic ACC who are encountered in the daily clinical practice. Our analysis confirms results from two previous published series (10, 11). Gem/Cape resulted a moderately active regimen with a clinical benefit rate (CBR) at 4 months of 30%, median PFS of 3 months, and median DSS of 8 months. CBR, PFS, and DSS observed in the present series were inferior to those obtained in the Sperone et al. trial (CBR 46%, median PFS 5.3 months, median DSS 9.8 months), while the Henning et al. series reported a lower CBR (20.8%) a similar PFS (median 3 months) and longer DSS (median 10 months) than the present study. Patient selection could account for the differences observed. Patterns and severity of toxicities were also comparable and overall indicate a good tolerability of this schedule. It is well known that PFS is influenced by treatment efficacy while survival mainly depends on disease aggressiveness and efficacy of subsequent treatment lines. We assumed here PFS as surrogate of Gem Cape efficacy. We found that, with the exception of surgery of the primary tumor (no-surgery HR 2.67, p.049), none of the most important ACC prognostic factors (including ENSAT stage, Ki67 value, hormonal hypersecretion) evaluated at initial diagnosis had any impact in predicting PFS of patients submitted to Gem/Cape chemotherapy and were not associated with DSS. Despite the number of previous chemotherapy lines failed to be associated with outcome, patients who received Gem/Cape after more than 19 months from diagnosis had a better DSS. It should be noted that patients addressed to a second-line therapy are a selected subset who have not died early or have not had a significant deterioration in performance status after the previous treatment lines and therefore represent a subset with better response rates and overall prognosis.

In this series, mitotane plasma concentration was not predictive of response and survival. Whether mitotane in association with chemotherapy should be continued or not beyond the first line is a matter of controversy (1). The absence of any predictive or prognostic role of plasma mitotane levels in our patient series, confirming a previous observation (11), supports the notion that the drug is not effective in this context and could be withdrawn. When considering patients clinical characteristics at Gem/Cape start, a more precise definition of those destined to have a poor outcome emerged. The pattern of metastatic sites showed a poor predictive value of PFS for lung, liver, and lymph nodal metastases at univariate analysis but not after adjusting for multiple comparisons. None of them showed to be prognostic for DSS. Surprisingly, the Charlson’s Comorbidity Index score ≥6 which defines patient’s vulnerabilities at baseline had no impact on PFS and DSS. Our result is in part in contrast with a much larger observation in all ACC patients from the US National Cancer Database in which a Charlson-Deyo comorbidity score >2 was associated with a poorer prognosis (19). On the other hand, the presence of tumor-associated symptoms (ECOG PS ≥1) was highly correlated with a poor PFS (HR 6.93, p.004). While the term “symptoms” is very generic, in our series the symptomatic patient often had pain, discomfort from organ compression or insufficiency, signs of anemia and of systemic inflammation (such as fever). The predictive role for PFS of PS might seem obvious and has wide consensus in clinical oncology. However, its meaning is to practically help the clinician to select patients that have a chance to obtain a benefit from chemotherapy sparing the others in which deterioration of QOL would be the inevitable result. Identification of laboratory parameters of sensitivity to one specific drug has traditionally failed in ACC patients regardless of the nature of the drug, chemotherapy, immunotherapy or molecular target-agent, as a consequence of a low frequency of highly responder patients and the rarity of the disease (20–22). Henning et al., investigated in ACC patients receiving Gem-based chemotherapy the prognostic and predictive role of the tissue expression of the human equilibrative nucleoside transporter type 1 (hENT1) and the subunit M1 of ribonucleotide reductase (RRM1), two enzymatic activities involved in response or resistance to Gemcitabine. Their results showed that both biomarkers were not useful as predictive markers of activity in patients receiving Gem-based chemotherapy (11). The current study additionally investigated the prognostic and predictive value of laboratory characteristics that can be easily found or calculated from clinical records. We found that presence of anemia, high neutrophils and low lymphocytes relative counts and the NLR ≥5 before the start of Gem/Cape chemotherapy were poor predictive factors for PFS at univariate analysis. Among them, only NLR maintained an independent predictive significance at multivariate analysis (HR 3.88, p.003). This observation is based on a very limited number of patients and has, therefore, a weak inferential power. NLR, however, has already shown to be a significant prognostic factor both in ACC (23, 24) and in other neoplasms (25, 26). Further interest of NLR in ACC derives from the fact that it may be increased from endogenous cortisol and/or from exogenous steroid replacement therapy that is a frequent condition in ACC patients (27). In our series NLR correlated with PFS but not with DSS thus appearing to be a predictive factor of Gem/Cape efficacy and not a prognostic factor. ALI and LIPI biomarkers have been described in patients with lung cancer (17, 18) and their role is still unknown in ACC patients. With regard to ALI biomarker, we found a predictive role in terms of PFS at univariate but not at multivariate analysis. Conversely, the LIPI index failed to be significantly associated with PSF and DSS.

In conclusion, the present analysis has some limitations linked to its retrospective nature and the limited number of patients. Nevertheless, it confirms the modest efficacy of Gem/Cape chemotherapy as second or further line of treatment for metastatic ACC patients. Gem/Cape should not be prescribed in patients with poor PS, rapidly progressing ACC and/or with high NLR as they are unlikely to obtain a benefit from this regimen. In line with this, it is unlikely that patients with newly diagnosed, full-blown ACC might derive significant benefit in first line. As Gemcitabine has potential as a demethylating agent and hypermethylation is a distinctive feature of aggressive ACC, in the future tumor methylation status could be evaluated as predictive factor of sensitivity to Gemcitabine. Further investigation is required to best integrate clinical and molecular data to address the correct ACC patient to the correct treatment.

## Data Availability Statement

The raw data supporting the conclusions of this article will be made available by the authors, without undue reservation.

## Ethics Statement

The studies involving human participants were reviewed and approved. The study was approved by the Institutional Ethical Review Board (ID: NP 3776/2019) and conducted in accordance with the principles of the Declaration of Helsinki. The patients/participants provided their written informed consent to participate in this study.

## Author Contributions

Conceptualization, AB. Methodology, SG. Software, AM, DC, and ML. Validation, SG, AB, and VF. Formal analysis, SG and AM. Investigation, SG, DC, ML, VF, BL, SS, and AB. Data curation, SG, AM, DC, ML, AV, and LF. Writing—original draft preparation, SG, AB. Writing—review and editing, SG and AB. Visualization, SG and ML. Supervision, AB and SS. Radiological assessment, RA. Project administration, AB. All authors contributed to the article and approved the submitted version.

## Funding

This work was granted by: AIRC project IG14411 (PI: AB), Fondazione Camillo Golgi, Brescia and Fondazione Internazionale di Ricerca in Medicina (F.I.R.M.) ONLUS, Cremona (Italy).

## Conflict of Interest

The authors declare that the research was conducted in the absence of any commercial or financial relationships that could be construed as a potential conflict of interest.
